# Evaluation of Awareness on Radiation Protection and Knowledge About Radiological Examinations in Healthcare Professionals Who Use Ionized Radiation at Work

**DOI:** 10.4274/mirt.00719

**Published:** 2014-06-05

**Authors:** Ayşegül Yurt, Berrin Çavuşoğlu, Türkan Günay

**Affiliations:** 1 Dokuz Eylül University Health Sciences Institute, Department of Medical Physics, İzmir, Turkey; 2 Dokuz Eylül University Faculty of Medicine, Department of the Public Health, İzmir, Turkey

**Keywords:** ionizing radiation, diagnostic imaging, radiation dosage, Radiation protection

## Abstract

**Objective:** In this study, we evaluated the knowledge and perception and mitigation of hazards involved in radiological examinations, focusing on healthcare personnel who are not in radiation-related occupations, but who use ionising radiation as a part of their work.

**Methods:** A questionnaire was applied to physicians, nurses, technicians and other staff working in different clinics that use radiation in their work, in order to evaluate their knowledge levels about ionizing radiation and their awareness about radiation doses resulting from radiological examinations. The statistical comparisons between the groups were analyzed with the Kruskal Wallis test using the SPSS program.

**Results:** Ninety two participants took part in the study. Their level of knowledge about ionizing radiation and doses in radiological examinations were found to be very weak. The number of correct answers of physicians, nurses, medical technicians and other personnel groups were 15.7±3.7, 13.0±4.0, 10.1±2.9 and 11.8±4.0, respectively. In the statistical comparison between the groups, the level of knowledge of physicians was found to be significantly higher than the level of the other groups (p=0.005).

**Conclusion:** The present study demonstrated that general knowledge in relation to radiation, radiation protection, health risks and doses used for radiological applications are insufficient among health professions using with ionizing radiation in their work.

## Abstract (tr)

**Amaç:** Bu çalışmada, radyasyon çalışanı olmayıp, iyonlaştırıcı radyasyonu işinin belli bir bölümünde kullanan, sağlık çalışanlarının iyonize radyasyon ışınlamaları ve dozları hakkındaki bilgilerinin ve algılarının değerlendirilmesi amaçlanmıştır.

**Yöntem:** İşlerinde radyasyon kullanan farklı kliniklerde çalışan doktor, hemşire, tekniker ve diğer personele, iyonize radyasyon hakkındaki bilgi düzeyleri ve radyolojik incelemeler sonucundaki radyasyon değerleri hakkındaki farkındalıklarını değerlendiren bir anket uygulandı. Gruplar arasındaki istatistiksel karşılaştırma SPSS istatistik programı kullanılarak Kruskal Wallis testi ile incelendi.

**Bulgular:** Çalışmada 92 katılımcı yer aldı. İyonize radyasyon ve radyolojik incelemelerdeki dozlar hakkındaki bilgi düzeylerinin oldukça zayıf olduğu görüldü. Doktor, hemşire, sağlık teknikeri/teknisyeni ve diğer personel gruplarının doğru cevap sayıları sırasıyla 15,7±3,7, 13,0±4,0, 10,1±2,9, 11,8±4,0 idi. Gruplar arasındaki istatistiksel karşılaştırmada doktorların bilgi düzeylerinin diğer gruplara göre anlamlı olarak daha yüksek olduğu bulundu (p=0,005).

**Sonuç:** Bu çalışma, işlerinde iyonlaştırıcı radyasyonu kullanan sağlık çalışanlarının radyolojik uygulamalarda kullanılan radyasyon, radyasyondan korunma, sağlık riskleri ve dozlarla ilgili genel bilginin yetersiz olduğunu gösterdi.

## INTRODUCTION

Radiation has negative biological effects on living organisms, which may vary depending on the dose and the duration of exposure (1,2). Despite some of the experimental and epidemiological studies (1), a threshold dose to cause cancer in humans has not yet been established. Since the doses of x-rays used for diagnostic purposes are small, it is generally considered that health risks to individuals are also small. However, the growing number of people exposed to x-ray radiation makes low-level x-ray radiation dosing a more pressing concern (3). 

X ray radiation has dose-dependent adverse effects that lead to an increased risk of developing cancers (4). Due to increases in the number of radiological examinations, as well as the doses used, the cancer risk in adults and children has been the focus of most studies (5). Although x-ray radiation for medical imaging is clinically useful, it is estimated that 20% of medical x-ray examinations are not beneficial, and that these and other unnecessary exposures leads to 100-250 cases of cancer each year in the UK (6). Although the risks are small for each individual, the large number of people exposed to x-ray radiation is expected to result in a significant number of related health problems in the future. In addition, it has been identified that healthcare personnel often do not have sufficient knowledge about the risks posed by x-ray exposure and the measures that should be taken to mitigate those risks (7). 

Several medical procedures, including angiography, fluoroscopy, computed tomography (CT) and radiographic imaging, utilise ionising radiation. The primary purpose of radiological imaging is to achieve the optimum quality image using the minimum possible dose. However, the dose limits permitted by international authorities may exceed in some interventional applications and in some cases (8). Therefore, it is extremely important to consider the safety of both the patient and the medical professional performing the procedure. 

In this study, we evaluated the knowledge and perception and mitigation of hazards involved in radiological examinations, focusing on healthcare personnel who are not in radiation-related occupations, but who use ionising radiation as a part of their work. 

## MATERIALS AND METHODS

In this study, target groups were healthcare personnels who use ionising radiation as a part of their work, and who work as an allied health personnel (nurses, technologist/technicians, other health professions) in different clinics. Healthcare personnel working in the departments of internal medicine (endoscopy unit), coronary angiography, orthopaedic surgery, brain surgery, intensive care unit, chest diseases, gastroenterology, urology, and the operating theatres were invited to participate in this study; 92 of them accepted. 

A questionnaire was given to these people to evaluate their knowledge of ionising radiation and their awareness of the radiation doses that result from radiological examinations. The participants were asked for details of personal information, including their age, gender, education level, occupation, and marital status, as well as professional information, including their area of expertise, how long they have worked, and whether or not they use ionising radiation at work. In addition, to evaluate their awareness of the radiation doses that result from examinations and measures to protect against ionising radiation, questions were asked relating to issues including the safe dose of ionising radiation in radiologic examinations, the average background radiation to which a person is subjected annually, the average effective dose for a standard chest x-ray for adults and the average effective radiation dose for a standard chest CT scan for an adult. Participants were asked to estimate the chest x-ray equivalent doses, rather than the exact radiation dose, for radiological applications that are commonly used. Participants were asked to complete 42 questions, and statistical analyses of the groupings were performed with the Kruskal-Wallis one-way analysis of variance using the SPSS; p<0.05 was deemed to indicate a significant difference. 

## RESULTS

Table 1 lists a summary of the gender, educational background, marital status and occupation of the participants. The participants considered that nuclear power stations, cosmic rays and medical services at hospitals to be the most common sources of ionising radiation; rock and soil, food intake, building materials were less well-known sources. More than the half of the participants reported that nuclear terrorism or nuclear weapons, as well as x-ray/CT applications, to be among their greatest concerns. Cancer was reported to be the largest single health risk associated with radiation, and approximately half of the group reported that this might also cause growth retardation in children. More than half (57.6%) of the participants reported that radiological examinations and applications may be performed for females who are likely to be pregnant if justified by the physician, and 44.6% of the respondents reported that radiological examinations are not to be performed on females who are likely to be pregnant. Only 8.7% of the respondents reported correctly that examinations must be conducted in accordance with the 10-day rule (Table 2). 

Only 21.6% of the participants reported that they considered the ionising radiation dose for general radiological applications to be moderately safe, and more than the half said that they had no knowledge of this matter. Of the respondents, 90.2% considered that human immunodeficiency virus (HIV) was the greatest health risk to which healthcare professionals may be exposed in the workplace, and 47% of participants stated that radiography and CT applications were significant health risks (Table 2). 

Only 7.6% of the respondents had any knowledge of the annual background radiation dose. Furthermore, only 13% were aware of the chest x-ray effective dose for adults, and only 4.3% of participants knew the standard thorax CT average effective dose value for adults. Of the participants, 1.1%-10.9% knew the doses of more complex applications with higher doses in comparison with the chest x-ray dose. Of the participants, 10.9% incorrectly stated that magnetic resonance imaging (MRI) and ultrasonography (US) entail a larger dose of radiation than a chest x-ray. No participants were able to correctly answer questions related to leg arteriograms, and the abdomen and chest x-ray dose equivalent of lumbar radiography (Table 3). The average number of correct answers of a total of 42 points was found to be greatest in the physicians (p=0.005) (Table 4). 

The issues relating to ionising radiation that participants mostly commonly reported that they would like to learn more about safety measures (80.4%), the safe dose of radiation (67.4%) and the action to be taken in the case of a radiation accident (53.3%). The first thought that participants reported on hearing the word “radiation” was Chernobyl (62%), cancer treatment (46.7%), Hiroshima (31.5%) and x-ray imaging (26.1%). Of the respondents, 97.6% reported that they would be concerned if they were to learn that they or their spouse was pregnant following a radiation examination, and 87% said that they would be concerned if their child or young nephews/nieces were required to undergo a radiological diagnosis or treatment.

## DISCUSSION

We found that the number of correct answers to questions related to the average background radiation dose that a person may be exposed to on an annual basis (~2.4 mSv), the dose from chest x-ray (~0.02-0.04 mSv), and the dose from a standard thorax CT application for adults (~3-9 mSv) revealed a basic lack of knowledge of the health risks from ionising radiation. In addition, the small numbers of correct responses for the chest x-ray dose equivalent doses for other radiological applications (Table 3) shows that the awareness of the relative health risks of different procedures is also poor. Furthermore, only 1 of 10 participants was able to give an answer for the dose evaluation of abdominal magnetic resonance imaging (MRI) and ultrasonography (US) of chest x-ray. It was a surprising result that the majority of respondents did not know that ionizing radiation is not used in MRI and US. 

In a survey performed by Zhou and colleagues (4), which was targeted at medical students and interns, it was found that 31.6% of participants correctly reported the dose received by patients during a standard chest x-ray, that only 11.3% were not aware of the fact that ionising radiation is not used in US and 25.5% did not know that ionising radiation is not used during MRI. Aslanoglu et al. reported that the knowledge of physicians and interns about radiation exposure is insufficien, and that 93.1% of the respondents did not know the radiation doses involved in radiological imaging procedures (2); furthermore, 4% stated that ionising radiation is used during US and 27.5% said that ionising radiation is used for MRI. 

A study by Shialkar et al. reported that 97% of physicians did not know the radiation doses received by patients during radiological investigations and that 5% claimed that ionising radiation is used during US and 8% claimed that ionising radiation is used in MRI (9). Jacobs et al. found that only 15%-29% of physicians knew the doses during chest x-ray examinations, and 10% stated that ionising radiation is used during US and 28% that ionising radiation is used for MRI (10). Quinn et al. reported that most physicians did not know the radiation doses received by patients during radiological procedures (6). In addition, they did not find a significant difference between those who were trained in radiation protection and those who were not. A report by Heyer et al. emphasised that the awareness of paediatricians about radiation doses and risks was greater than that of physicians in other hospital departments (11). A survey by Keijzers and Britton found that emergency doctors had a variable knowledge of the risks from radiation exposure, which overall was poor (12). 

In a study of nurses working in a radiology department in Kuwait carried out by Alotaibi et al., it was found that a majority of participants had no knowledge of radiation protection measures and that they were not knowledgeable about the risks of radiation (13). These nurses stated that they were concerned about radiation and would like to learn more about health risks associated with radiation. 

This lack of knowledge of the safety issues associated with ionising radiation is in overall agreement with a number of other reports (2,4,6,9,10,11,12,13). This lack of knowledge means that the healthcare professionals are unable to effectively protect either themselves or their patients from ionising radiation. In Turkey, no standard courses on radiation safety for health professionals exist. However, courses on radiation, the biological effects of radiation, and radiation protection should be included in the educational curriculum of health professionals (including nurses and medical technicians). Physicians should encounter these topics in radiology courses. In our study, the fact that the number of correct answers given by physicians was higher than by the other groups may be because courses on radiology are included in most medical degrees. 

Based on the results reported here, it appears that improved education planning for healthcare professionals into safety measures associated with ionising radiation in required. As a result of studies and regulations in the UK (7,8,14), there is a consensus that radiation safety should be taught to medical students, and a formal structure that includes radiology as a core curriculum subject has been created. The Turkish Atomic Energy Agency offers a course on ionising radiation, health risks, and the dosage ranges used in certain procedures. The Ministry of Health should require health professionals who use ionising radiation as a part of their work to complete this course. 

Healthcare professionals working with ionising radiation should be provided with an educational program on doses per application, a risk/benefit analysis, the necessity of medical exposure, and the biological effects of radiation. In addition, an obligatory radiation safety course should be provided at medical schools, as well as postgraduate radiation protection and radiation safety training. A series of studies of the reasons for, and quantities of, unnecessary radiological imaging techniques requested by physicians is expected to be beneficial in reducing the number of patients exposed to potentially harmful ionising radiation. 

## CONCLUSION

We have described the results of a survey on the safety issues related to the use of radiation for medical procedures which is designed to examine the knowledge of healthcare professionals who are not radiation professionals but do use ionising radiation as part of their work. We find that an awareness of the health risks associated with ionising radiation is lacking, and furthermore, that this is in general agreement with the results of other similar surveys. Courses on radiatio, and the biological effects of radiation should be included in the training of healthcare professionals, both during and after their education, to increase awareness of the safety protocols required to protect from the hazardous effects of ionising radiation.

## Figures and Tables

**Table 1:**
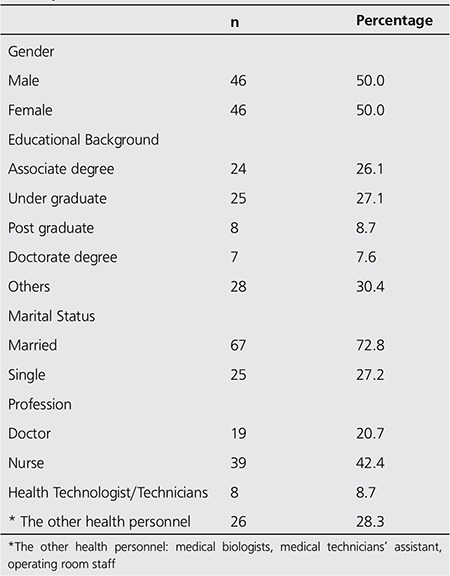
Distribution of study participants according to descriptive features

**Table 2:**
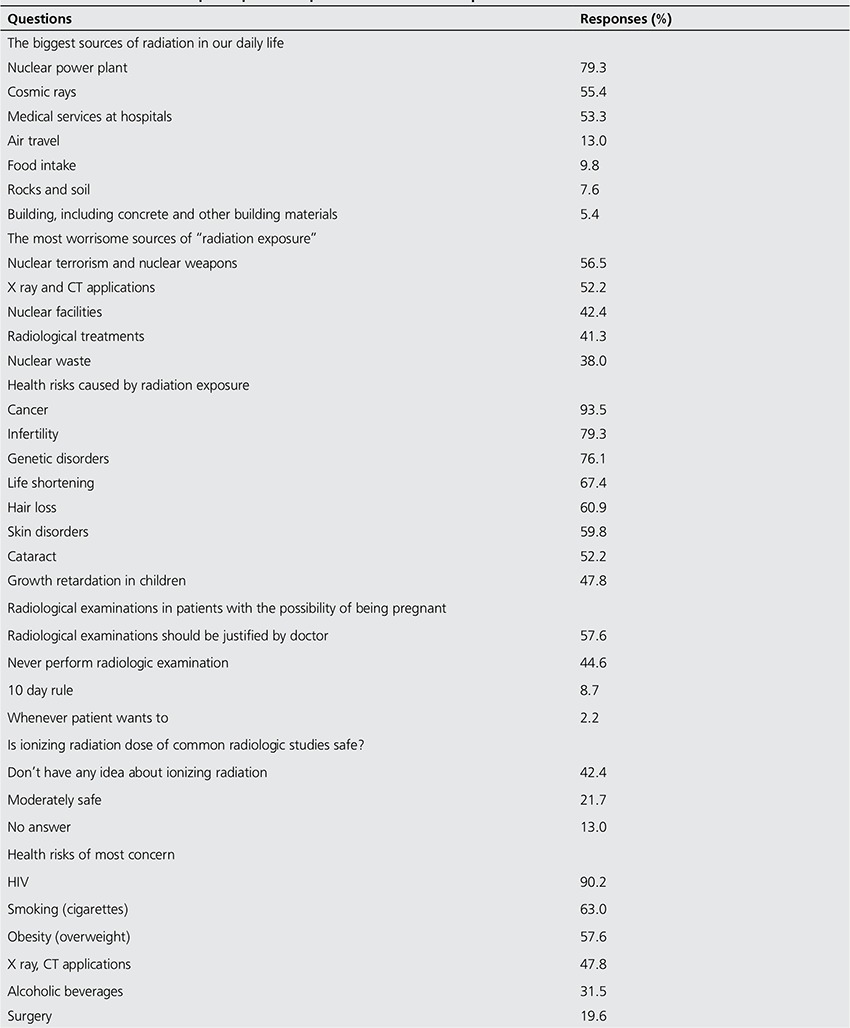
The distribution of participants’ responses to informative questions

**Table 3:**
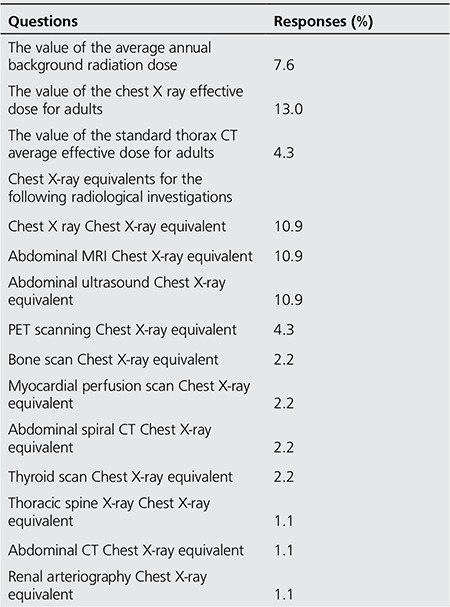
The distribution of participants’ responses to informative questions related to radiation dose

**Table 4:**
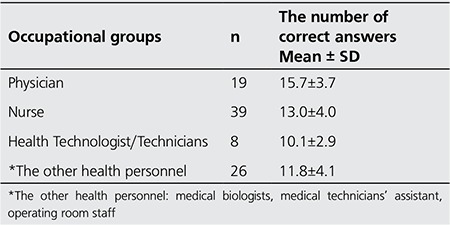
The number of correct answers according to the occupational groups
